# 4-{[1-(4-Bromo­phen­yl)eth­yl]amino­meth­yl}phenol

**DOI:** 10.1107/S1600536811028339

**Published:** 2011-07-23

**Authors:** Karilys Gonzalez Nieves

**Affiliations:** a Department of Chemistry, University of Puerto Rico, San Juan, PR 00931, Puerto Rico

## Abstract

The title compound, C_15_H_16_BrNO, obtained from a two-step reaction, was prepared for use in transition metal chemistry as a phenolic ligand with bulky substituents. Inter­molecular N—H⋯O and O—H⋯N hydrogen bonds are present in the crystal structure.

## Related literature

For chirality induction in metal complexes, see: Fan *et al.* (2010 ▶); Amendola *et al.* (2010 ▶). For imine reduction, see: Menta & Prabhakar (1995 ▶).
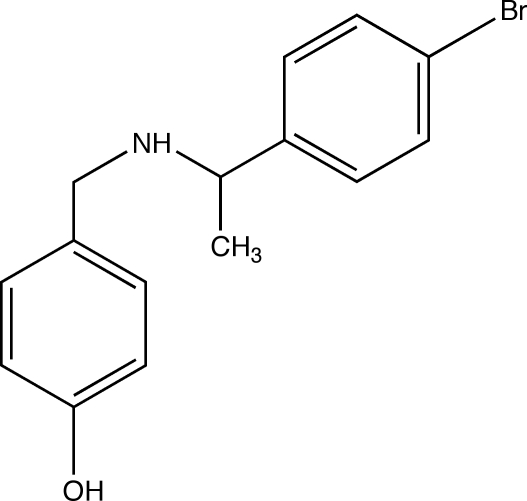


## Experimental

### 

#### Crystal data

C_15_H_16_BrNO
*M* *_r_* = 306.20
Monoclinic, 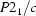

*a* = 12.1753 (10) Å
*b* = 8.1939 (7) Å
*c* = 14.0326 (11) Å
β = 93.333 (1)°
*V* = 1397.6 (2) Å^3^
*Z* = 4
Mo *K*α radiation
μ = 2.93 mm^−1^
*T* = 296 K
0.20 × 0.16 × 0.14 mm

#### Data collection

Bruker APEXII CCD diffractometer
Absorption correction: multi-scan (*SADABS*; Sheldrick, 2008*a* ▶) *T*_min_ = 0.592, *T*_max_ = 0.685
14961 measured reflections
3094 independent reflections
2119 reflections with *I* > 2σ(*I*)
*R*_int_ = 0.031

#### Refinement

*R*[*F* ^2^ > 2σ(*F* ^2^)] = 0.031
*wR*(*F* ^2^) = 0.077
*S* = 1.00
3094 reflections
168 parameters
H atoms treated by a mixture of independent and constrained refinement
Δρ_max_ = 0.25 e Å^−3^
Δρ_min_ = −0.40 e Å^−3^

### 

Data collection: *APEX2* (Bruker, 2005 ▶); cell refinement: *SAINT* (Bruker, 1999 ▶); data reduction: *SAINT*; program(s) used to solve structure: *SHELXS97* (Sheldrick, 2008*b* ▶); program(s) used to refine structure: *SHELXL97* (Sheldrick, 2008*b* ▶); molecular graphics: *SHELXTL* (Sheldrick, 2008 ▶); software used to prepare material for publication: *SHELXTL*.

## Supplementary Material

Crystal structure: contains datablock(s) I, global. DOI: 10.1107/S1600536811028339/fy2015sup1.cif

Supplementary material file. DOI: 10.1107/S1600536811028339/fy2015Isup2.cml

Structure factors: contains datablock(s) I. DOI: 10.1107/S1600536811028339/fy2015Isup3.hkl

Additional supplementary materials:  crystallographic information; 3D view; checkCIF report

## Figures and Tables

**Table 1:** Hydrogen-bond geometry (Å, °)

*D*—H⋯*A*	*D*—H	H⋯*A*	*D*⋯*A*	*D*—H⋯*A*
O1—H1*A*⋯N1^i^	0.82	2.05	2.794 (2)	150
N1—H1⋯O1^ii^	0.75 (2)	2.40 (2)	3.144 (3)	168 (2)

Symmetry codes: (i) 
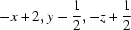
; (ii) 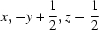.

**Fig. 1.**
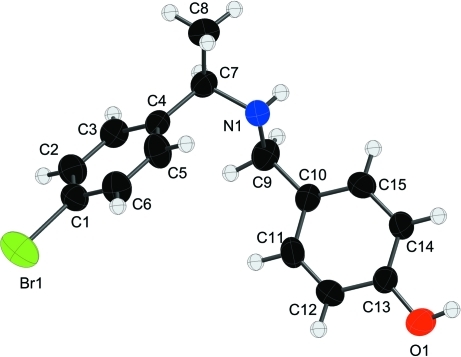
Molecular structure of compound (I) with 50% probability displacement ellipsoid for non hydrogen atoms.

**Fig. 2.**
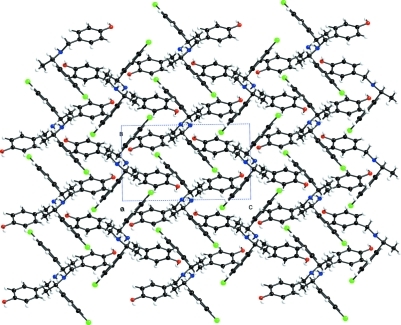
Packing diagram of compound (I).

**Fig. 3.**
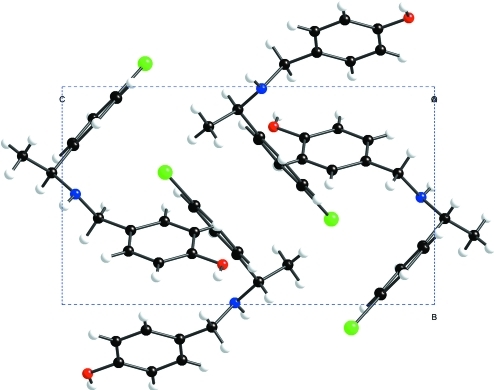
Molecular view along a axis.

**Fig. 4.**
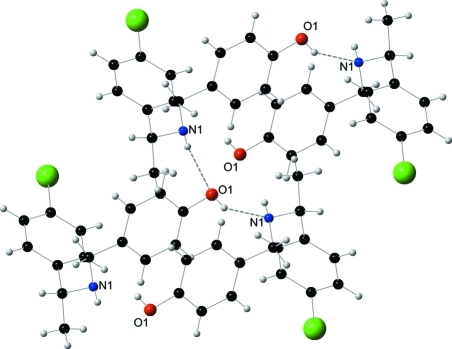
Molecular structure of compound (I) with hydrogen bonding.

## supplementary crystallographic information

### Comment

The title compound was prepared to be used as a ligand in order to induce chirality in transition metal complexes in combination with other bridging ligands. Here, we report the crystal structure of racemic 4-((1-(4-bromophenyl)ethylamino)methyl)phenol. In the crystal structure (Fig. 4), intermolecular hydrogen bonding was observed between N1—H1···O1 and O1—H1A···N1 (Table 1).

### Experimental

An excess of racemic 4-bromo-α-methylbenzylamine (0.069 g, 0.45 mmol) was added to a solution of 4-hydroxybenzaldehyde (0.036 g, 0.29 mmol) in ethyl acetate. The solution was stirred overnight at room temperature. The solvent was removed under vacuum to obtain 4-((1-(4-bromophenyl)ethylimino)methyl)phenol as a white solid. The Schiff base was dissolved in anhydrous MeOH and an excess of NaBH_4_ was added in several portions to the reaction mixture. After 24 h, water was added to the solution, and stirring was continued for two more hours. Methanol was removed under vacuum and the remaining aqueous phase was extracted three times with ethyl acetate. The combinated organic extracts were dried with magnesium sulfate, and the solvent was removed under vacuum to obtain a white solid. The product was dissolved in acetone and crystals were obtained by vapor diffusion of pentane.

### Refinement

All non-H atoms were refined anisotropically. H atoms were positioned geometrically (except the H on the N), with C—H = 0.96 (CH_3_), 0.97 (CH_2_), 0.98 (CH) and 0.93 (aromatic CH) Å, O—H = 0.82 Å and constrained with *U*_iso_(H) = 1.5 *U*_eq_(parent) for methyl H and O—H and *U*_iso_(H) = 1.2 *U*_eq_(parent) for all other H atoms. The H on the N atom was generated with N—H = 0.87 Å, *U*_iso_(H) = 0.033.

### Crystal data

**Table:**

C_15_H_16_BrNO	*F*(000) = 624
*M**_r_* = 306.20	*D*_x_ = 1.455 Mg m^−^^3^
Monoclinic, *P*2_1_/*c*	Mo *K*α radiation, λ = 0.71073 Å
Hall symbol: -P 2ybc	Cell parameters from 5896 reflections
*a* = 12.1753 (10) Å	θ = 2.3–26.2°
*b* = 8.1939 (7) Å	µ = 2.93 mm^−^^1^
*c* = 14.0326 (11) Å	*T* = 296 K
β = 93.333 (1)°	Block, colourless
*V* = 1397.6 (2) Å^3^	0.20 × 0.16 × 0.14 mm
*Z* = 4	

### Data collection

**Table:**

Bruker APEXII CCD diffractometer	3094 independent reflections
Radiation source: fine-focus sealed tube	2119 reflections with *I* > 2σ(*I*)
graphite	*R*_int_ = 0.031
φ and ω scans	θ_max_ = 27.2°, θ_min_ = 2.9°
Absorption correction: multi-scan (*SADABS*; Sheldrick, 2008a)	*h* = −15→15
*T*_min_ = 0.592, *T*_max_ = 0.685	*k* = −10→10
14961 measured reflections	*l* = −17→18

### Refinement

**Table:**

Refinement on *F*^2^	Primary atom site location: structure-invariant direct methods
Least-squares matrix: full	Secondary atom site location: difference Fourier map
*R*[*F*^2^ > 2σ(*F*^2^)] = 0.031	Hydrogen site location: inferred from neighbouring sites
*wR*(*F*^2^) = 0.077	H atoms treated by a mixture of independent and constrained refinement
*S* = 1.00	*w* = 1/[σ^2^(*F*_o_^2^) + (0.031*P*)^2^ + 0.5405*P*] where *P* = (*F*_o_^2^ + 2*F*_c_^2^)/3
3094 reflections	(Δ/σ)_max_ = 0.002
168 parameters	Δρ_max_ = 0.25 e Å^−^^3^
0 restraints	Δρ_min_ = −0.40 e Å^−^^3^

### Special details

**Table:**

Experimental. IR (KBr cm^-1^): 3448 (*m*), 3280 (*m*), 2970 (*m*), 2824 (*m*), 1613 (m, C=C), 1592 (*m*), 1516 (*s*), 1469 (*m*), 1373 (*m*), 1251 (*s*), 1174 (*m*), 1009 (*s*), 862 (w), 829 (*s*), 635 (w), 501 (w). ^1^H-NMR (500 MHz, d_6_-acetone) p.p.m.: 1.28–1.30 (d, 3H, CH_3_), 3.44–3.52 (dd, 2H, –CH_2_NH), 3.77–3.81 (m, 1H, CHCH_3_), 6.75–6.77 (d, 2H, aromatic protons in the phenyl ring), 7.10–7.11 (d, 2H, aromatic protons in bromophenyl ring), 7.35–7.37 (d, 2H, aromatic protons in bromophenyl ring), 7.48–7.50 (d, 2H, aromatic protons in the phenyl ring). ^13^C-NMR (125 MHz, d_6_-acetone) p.p.m.: 24.7 (CH_3_CH–), 51.5 (–CH_2_NH), 57.5 (–CHCH_3_), 115.8 and 132.1 (aromatic carbons in the phenyl ring), 132.1 (quaternary carbon), 120.6 (quaternary carbon, Br), 129.7 and 130.1 (aromatic carbons in the bromophenyl ring), 157.1 (quaternary carbon, OH).
Geometry. All e.s.d.'s (except the e.s.d. in the dihedral angle between two l.s. planes) are estimated using the full covariance matrix. The cell e.s.d.'s are taken into account individually in the estimation of e.s.d.'s in distances, angles and torsion angles; correlations between e.s.d.'s in cell parameters are only used when they are defined by crystal symmetry. An approximate (isotropic) treatment of cell e.s.d.'s is used for estimating e.s.d.'s involving l.s. planes.
Refinement. Refinement of *F*^2^ against ALL reflections. The weighted *R*-factor *wR* and goodness of fit *S* are based on *F*^2^, conventional *R*-factors *R* are based on *F*, with *F* set to zero for negative *F*^2^. The threshold expression of *F*^2^ > σ(*F*^2^) is used only for calculating *R*-factors(gt) *etc*. and is not relevant to the choice of reflections for refinement. *R*-factors based on *F*^2^ are statistically about twice as large as those based on *F*, and *R*- factors based on ALL data will be even larger.

### Fractional atomic coordinates and isotropic or equivalent isotropic displacement parameters (Å^2^)

**Table:**

	*x*	*y*	*z*	*U*_iso_*/*U*_eq_	
Br1	0.50613 (2)	1.10683 (4)	0.22306 (2)	0.07679 (13)	
C1	0.57389 (19)	0.9552 (3)	0.14304 (16)	0.0493 (5)	
C2	0.51175 (19)	0.8545 (3)	0.08329 (17)	0.0548 (6)	
H2	0.4354	0.8616	0.0801	0.066*	
C3	0.56405 (18)	0.7419 (3)	0.02779 (16)	0.0491 (5)	
H3	0.5220	0.6728	−0.0123	0.059*	
C4	0.67743 (17)	0.7301 (3)	0.03077 (15)	0.0433 (5)	
C5	0.73784 (19)	0.8363 (3)	0.09060 (18)	0.0585 (6)	
H5	0.8143	0.8322	0.0927	0.070*	
C6	0.6869 (2)	0.9475 (3)	0.14675 (18)	0.0574 (6)	
H6	0.7285	1.0170	0.1869	0.069*	
C7	0.73506 (19)	0.6046 (3)	−0.02810 (15)	0.0477 (5)	
H7	0.6796	0.5310	−0.0577	0.057*	
C8	0.7982 (2)	0.6822 (3)	−0.10623 (18)	0.0675 (7)	
H8A	0.8519	0.7565	−0.0783	0.101*	
H8B	0.7481	0.7406	−0.1492	0.101*	
H8C	0.8346	0.5988	−0.1406	0.101*	
C9	0.75156 (18)	0.3972 (3)	0.09742 (17)	0.0533 (6)	
H9A	0.7273	0.3024	0.0606	0.064*	
H9B	0.6866	0.4527	0.1178	0.064*	
C10	0.81988 (17)	0.3415 (3)	0.18429 (15)	0.0433 (5)	
C11	0.78927 (19)	0.3829 (3)	0.27448 (17)	0.0533 (6)	
H11	0.7270	0.4470	0.2805	0.064*	
C12	0.8484 (2)	0.3320 (3)	0.35547 (17)	0.0567 (6)	
H12	0.8266	0.3631	0.4152	0.068*	
C13	0.94031 (17)	0.2344 (3)	0.34832 (16)	0.0464 (5)	
C14	0.97270 (17)	0.1923 (3)	0.25915 (16)	0.0511 (6)	
H14	1.0348	0.1279	0.2533	0.061*	
C15	0.91298 (18)	0.2458 (3)	0.17840 (16)	0.0518 (6)	
H15	0.9359	0.2168	0.1186	0.062*	
N1	0.81141 (15)	0.5077 (2)	0.03586 (14)	0.0449 (4)	
O1	0.99339 (13)	0.1835 (2)	0.43153 (11)	0.0617 (4)	
H1A	1.0488	0.1325	0.4195	0.093*	
H1	0.8475 (17)	0.456 (3)	0.0064 (14)	0.033 (6)*	

### Atomic displacement parameters (Å^2^)

**Table:**

	*U*^11^	*U*^22^	*U*^33^	*U*^12^	*U*^13^	*U*^23^
Br1	0.0736 (2)	0.0705 (2)	0.0891 (2)	0.00063 (14)	0.02858 (15)	−0.02241 (16)
C1	0.0551 (13)	0.0417 (12)	0.0522 (13)	0.0010 (10)	0.0123 (11)	0.0010 (11)
C2	0.0435 (12)	0.0577 (15)	0.0637 (15)	−0.0006 (11)	0.0079 (11)	−0.0007 (12)
C3	0.0467 (12)	0.0487 (13)	0.0512 (13)	−0.0049 (10)	−0.0029 (10)	−0.0018 (11)
C4	0.0472 (12)	0.0412 (11)	0.0411 (12)	0.0027 (10)	−0.0006 (9)	0.0042 (10)
C5	0.0421 (12)	0.0566 (14)	0.0763 (17)	0.0020 (11)	−0.0012 (12)	−0.0133 (13)
C6	0.0556 (14)	0.0496 (13)	0.0665 (16)	−0.0027 (11)	−0.0014 (12)	−0.0107 (12)
C7	0.0506 (12)	0.0489 (12)	0.0427 (12)	0.0025 (10)	−0.0040 (10)	0.0009 (11)
C8	0.0771 (18)	0.0749 (18)	0.0510 (14)	0.0131 (15)	0.0094 (13)	0.0074 (13)
C9	0.0430 (12)	0.0539 (13)	0.0626 (15)	−0.0028 (11)	−0.0008 (11)	0.0085 (12)
C10	0.0399 (11)	0.0394 (11)	0.0506 (13)	−0.0017 (9)	0.0035 (10)	0.0084 (10)
C11	0.0475 (13)	0.0493 (13)	0.0641 (15)	0.0123 (10)	0.0108 (11)	0.0055 (12)
C12	0.0605 (15)	0.0623 (15)	0.0489 (14)	0.0114 (12)	0.0153 (12)	0.0037 (12)
C13	0.0433 (11)	0.0479 (12)	0.0483 (13)	0.0001 (10)	0.0070 (10)	0.0112 (10)
C14	0.0435 (12)	0.0584 (14)	0.0524 (14)	0.0099 (10)	0.0107 (11)	0.0078 (11)
C15	0.0477 (13)	0.0623 (15)	0.0462 (13)	0.0050 (11)	0.0091 (10)	0.0009 (11)
N1	0.0432 (10)	0.0470 (11)	0.0448 (11)	0.0061 (9)	0.0044 (9)	0.0020 (9)
O1	0.0559 (10)	0.0796 (12)	0.0501 (9)	0.0145 (9)	0.0084 (8)	0.0167 (9)

### Geometric parameters (Å, °)

**Table:**

Br1—C1	1.895 (2)	C9—N1	1.473 (3)
C1—C6	1.375 (3)	C9—C10	1.506 (3)
C1—C2	1.372 (3)	C9—H9A	0.9700
C2—C3	1.386 (3)	C9—H9B	0.9700
C2—H2	0.9300	C10—C11	1.382 (3)
C3—C4	1.382 (3)	C10—C15	1.384 (3)
C3—H3	0.9300	C11—C12	1.375 (3)
C4—C5	1.390 (3)	C11—H11	0.9300
C4—C7	1.516 (3)	C12—C13	1.384 (3)
C5—C6	1.376 (3)	C12—H12	0.9300
C5—H5	0.9300	C13—O1	1.366 (3)
C6—H6	0.9300	C13—C14	1.377 (3)
C7—N1	1.484 (3)	C14—C15	1.382 (3)
C7—C8	1.515 (3)	C14—H14	0.9300
C7—H7	0.9800	C15—H15	0.9300
C8—H8A	0.9600	N1—H1	0.75 (2)
C8—H8B	0.9600	O1—H1A	0.8200
C8—H8C	0.9600		
			
C6—C1—C2	120.7 (2)	H8B—C8—H8C	109.5
C6—C1—Br1	118.46 (18)	N1—C9—C10	113.10 (18)
C2—C1—Br1	120.85 (18)	N1—C9—H9A	109.0
C1—C2—C3	119.3 (2)	C10—C9—H9A	109.0
C1—C2—H2	120.4	N1—C9—H9B	109.0
C3—C2—H2	120.4	C10—C9—H9B	109.0
C4—C3—C2	121.3 (2)	H9A—C9—H9B	107.8
C4—C3—H3	119.3	C11—C10—C15	117.3 (2)
C2—C3—H3	119.3	C11—C10—C9	120.04 (19)
C3—C4—C5	117.8 (2)	C15—C10—C9	122.6 (2)
C3—C4—C7	121.6 (2)	C12—C11—C10	121.7 (2)
C5—C4—C7	120.55 (19)	C12—C11—H11	119.1
C6—C5—C4	121.3 (2)	C10—C11—H11	119.1
C6—C5—H5	119.3	C11—C12—C13	120.2 (2)
C4—C5—H5	119.3	C11—C12—H12	119.9
C1—C6—C5	119.5 (2)	C13—C12—H12	119.9
C1—C6—H6	120.3	O1—C13—C14	123.6 (2)
C5—C6—H6	120.3	O1—C13—C12	117.3 (2)
N1—C7—C4	109.06 (17)	C14—C13—C12	119.1 (2)
N1—C7—C8	109.62 (19)	C13—C14—C15	120.1 (2)
C4—C7—C8	112.33 (19)	C13—C14—H14	120.0
N1—C7—H7	108.6	C15—C14—H14	120.0
C4—C7—H7	108.6	C10—C15—C14	121.6 (2)
C8—C7—H7	108.6	C10—C15—H15	119.2
C7—C8—H8A	109.5	C14—C15—H15	119.2
C7—C8—H8B	109.5	C9—N1—C7	111.68 (17)
H8A—C8—H8B	109.5	C9—N1—H1	107.4 (16)
C7—C8—H8C	109.5	C7—N1—H1	109.6 (16)
H8A—C8—H8C	109.5	C13—O1—H1A	109.5
			
C6—C1—C2—C3	1.3 (4)	N1—C9—C10—C15	−64.5 (3)
Br1—C1—C2—C3	−178.17 (17)	C15—C10—C11—C12	0.1 (3)
C1—C2—C3—C4	−0.6 (3)	C9—C10—C11—C12	178.9 (2)
C2—C3—C4—C5	−0.7 (3)	C10—C11—C12—C13	−1.0 (4)
C2—C3—C4—C7	178.6 (2)	C11—C12—C13—O1	−177.9 (2)
C3—C4—C5—C6	1.4 (4)	C11—C12—C13—C14	1.4 (4)
C7—C4—C5—C6	−178.0 (2)	O1—C13—C14—C15	178.5 (2)
C2—C1—C6—C5	−0.7 (4)	C12—C13—C14—C15	−0.8 (4)
Br1—C1—C6—C5	178.81 (19)	C11—C10—C15—C14	0.5 (3)
C4—C5—C6—C1	−0.7 (4)	C9—C10—C15—C14	−178.2 (2)
C3—C4—C7—N1	−126.1 (2)	C13—C14—C15—C10	−0.2 (4)
C5—C4—C7—N1	53.2 (3)	C10—C9—N1—C7	−160.10 (19)
C3—C4—C7—C8	112.2 (2)	C4—C7—N1—C9	71.1 (2)
C5—C4—C7—C8	−68.5 (3)	C8—C7—N1—C9	−165.5 (2)
N1—C9—C10—C11	116.7 (2)		

### Hydrogen-bond geometry (Å, °)

**Table:**

*D*—H···*A*	*D*—H	H···*A*	*D*···*A*	*D*—H···*A*
O1—H1A···N1^i^	0.82	2.05	2.794 (2)	150.
N1—H1···O1^ii^	0.75 (2)	2.40 (2)	3.144 (3)	168 (2)

Symmetry codes: (i) −*x*+2, *y*−1/2, −*z*+1/2; (ii) *x*, −*y*+1/2, *z*−1/2.
